# Supplementary material for: IFITM3 Clusters on Virus Containing Endosomes and Lysosomes Early in the Influenza A Infection of Human Airway Epithelial Cells
Source: Viruses. 2019 Jun 12;11(6):548. doi: 10.3390/v11060548 (PMC6631848; doi:10.3390/v11060548)
Supplement: Supplementary file 1 [file viruses-11-00548-s001.pdf]

Figure S1

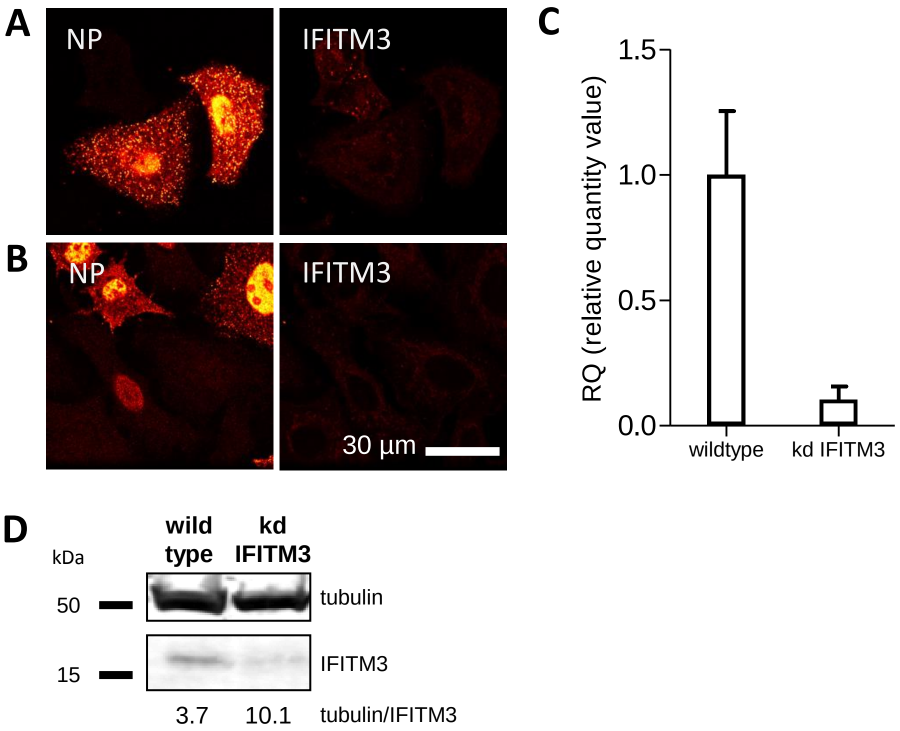

A
NP
IFITM3
B
NP
IFITM3
30 µm
C
1.5
1.0
0.5
0.0
RQ (relative quantity value)
wildtype
kd IFITM3
D
kDa
wild type
kd IFITM3
50
15
tubulin
IFITM3
3.7
10.1
tubulin/IFITM3

Figure S2

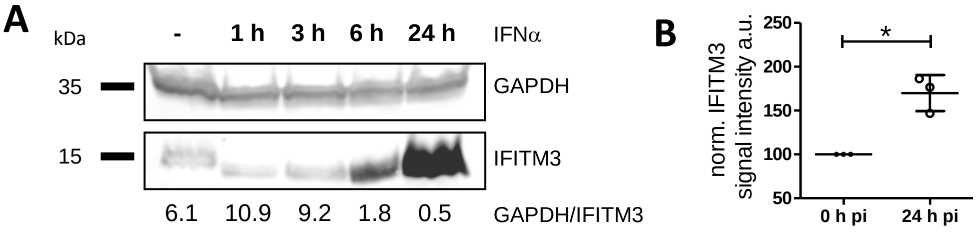
A
kDa
-
1 h
3 h
6 h
24 h
IFNα
35
GAPDH
15
IFITM3
6.1
10.9
9.2
1.8
0.5
GAPDH/IFITM3
B
norm. IFITM3 signal intensity a.u.
250
200
150
100
50
*
0 h pi
24 h pi

Figure S3

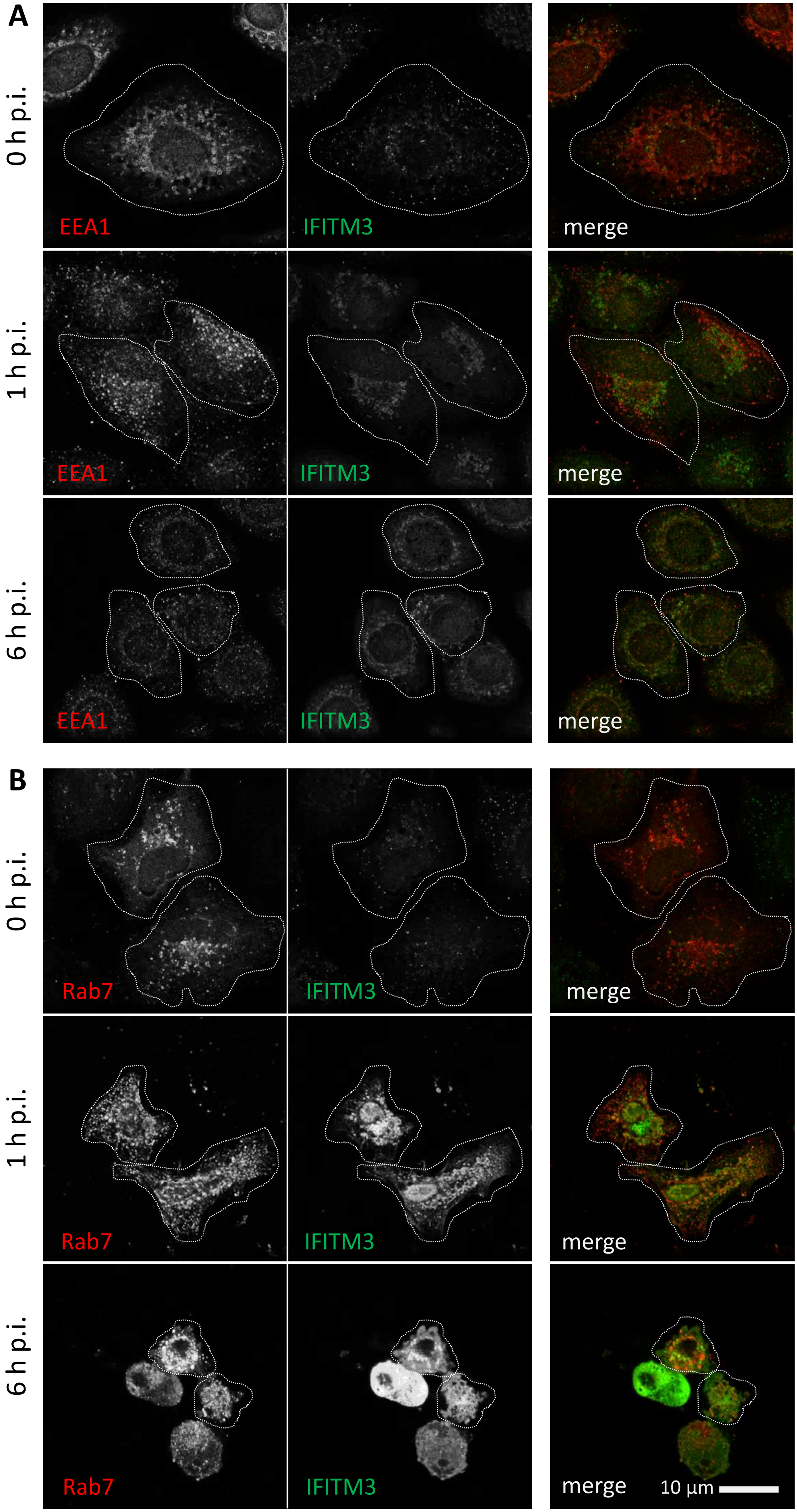

Figure S3

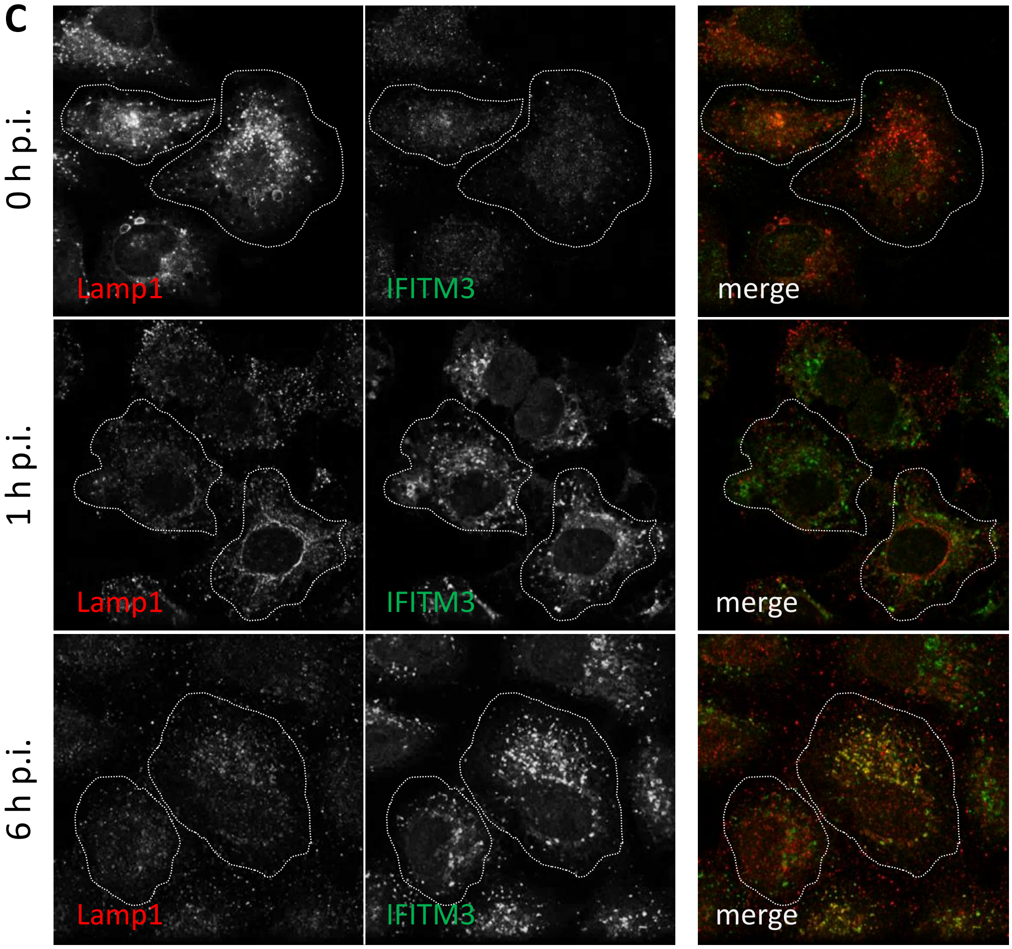

C
0 h p.i.
1 h p.i.
6 h p.i.
Lamp1
IFITM3
merge
Lamp1
IFITM3
merge
Lamp1
IFITM3
merge

Figure S4

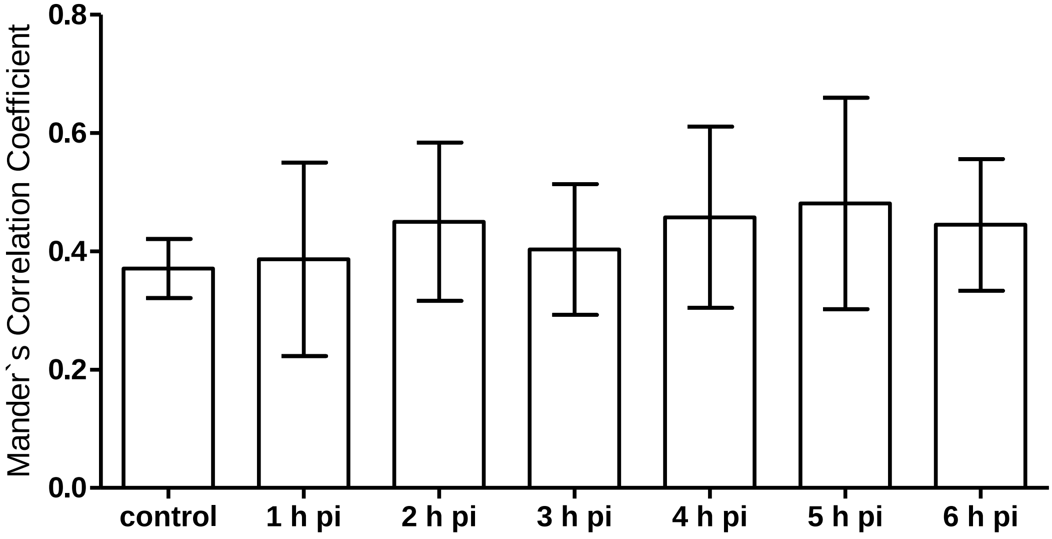

Mander`s Correlation Coefficient
0.8
0.6
0.4
0.2
0.0
control
1 h pi
2 h pi
3 h pi
4 h pi
5 h pi
6 h pi

Figure S5

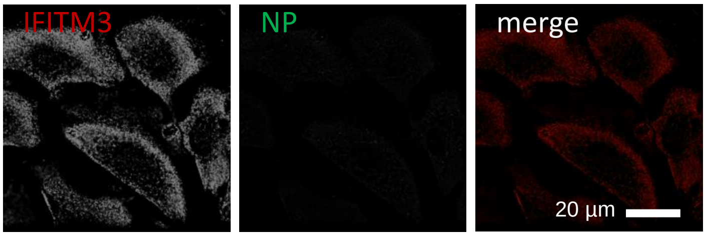

Supplementary figure legends

Figure S1. CRISPR/Cas mediated knock-down of IFITM3 in A549 results in decreased levels of IFITM3 protein. A549 wildtype **(A)** and IFITM3 knock-down **(B)** cells subjected for immunofluorescence were infected with IAV A/Hong Kong/1/1968 (MOI = 1) and fixed at 8 h pi. Cells were stained for IFITM3 and anti-nucleoprotein (NP). Images show representations of productively infected (NP signals inside the nucleus) and non-infected (no NP signals) A549 cells. Image acquisition was performed using 60 nm (= confocal mode) pixel size. **(C)** Quantitative Real-time PCR with IFITM3 specific primer sets validated knock-down in A549 cells. **(D)** Immunoblot analysis of IFITM3 levels in whole-cell lysates from A549 wildtype and IFITM3 knock-down cells. Tubulin was used as loading control. Panels are derived from a single cropped blot with lanes 1 and 2 belonging to the same membrane and from the same experiment.

Figure S2. IFITM3 abundance increases upon hIFNα induction and IAV infection at 24 h pi in A549 cells. **(A)** IFITM3 abundance in the absence and presence of 100 U/ml hIFNα for the indicated times was determined by western blotting. GAPDH level was used as loading control. The relative expression of IFITM3 was calculated as ration between the GAPDH and IFITM3 signals. Panels are derived from the same cropped blots with all lanes belonging to a single membrane and coming from the same experiment. Signal intensities were determined using the Li-Cor Odyssey Software. **(B)** Quantitative analysis of signal intensities of IFITM3 levels based on immunoblots of whole-cell lysates from non-infected (0 h pi) and A/Hong Kong/1/1968 infected (24 h pi) A549 wildtype cells. Tubulin was used as loading control. Mean values of three independent experiments are plotted as mean (SD).

Figure S3. IFITM3 proteins cover early, late and recycling endosomes at distinct time points post infection. Indirect immunofluorescence analysis using anti-IFITM3 antibody (green) in combination with

(A) anti-EEA1 antibody (red,), **(B)** anti-Rab7 antibody (red) and **(C)** anti-Lamp1 antibody (red). A549 cells subjected for fluorescence microscopy were infected with IAV A/Hong Kong/1/1968 (MOI = 1) and fixed with ice cold methanol at **(i)** 0 h pi (control), **(ii)** 1 h pi, and **(iii)** 6 h pi. Images shown represent the major subcellular distribution of each protein. The periphery of the immunostained cells was outlined with white dot lines on the basis of the cell morphology. Image acquisition was performed using 60 nm (= confocal mode) pixel size.

Figure S4. IFITM3 abundance stays constant in recycling endosomes up to 6 h pi in IAV infected A549 cells. Signal intensities for IFITM3 were correlated to the recycling endosomal marker, Rab11. Mander`s Correlation Coefficients from three independent experiments for each condition were calculated using the coloc2 plugin implemented in Fiji software. Mean values are plotted as mean (SD).

Figure S5. Indirect immunofluorescence analysis of naïve human small airway epithelial cells (HSAEpCs) using anti-IFITM3 (red) and anti-NP (green). Image acquisition was performed using 60 nm pixel size.
